# Characterization of Feruloyl Esterases Produced by the Four *Lactobacillus* Species: *L. amylovorus*, *L. acidophilus*, *L. farciminis* and *L. fermentum*, Isolated from Ensiled Corn Stover

**DOI:** 10.3389/fmicb.2017.00941

**Published:** 2017-06-02

**Authors:** Zhenshang Xu, Huiying He, Susu Zhang, Tingting Guo, Jian Kong

**Affiliations:** State Key Laboratory of Microbial Technology, Shandong UniversityJinan, China

**Keywords:** silage, *Lactobacillus*, feruloyl esterase, hydroxycinnamic esters, hydroxycinnamic acids

## Abstract

Lactic acid bacteria (LAB) play important roles in silage fermentation, which depends on the production of sufficient organic acids to inhibit the growth of undesirable microorganisms. However, LAB are not able to degrade cellulose and hemicellulose. Bacteria and fibrolytic enzymes are usually used as inoculants to improve the silage quality and digestibility. In the present study, we isolated four *Lactobacillus* strains (*L. amylovorus* CGMCC 11056, *L. acidophilus* CCTCC AB2010208, *L. farciminis* CCTCC AB2016237 and *L. fermentum* CCTCC AB2010204) with feruloyl esterase (FAE) activities from ensiled corn stover (CS) by a plate screening assay. The genes encoding FAEs were cloned and hetero-expressed in *Escherichia coli*. The optimal temperature and pH of these purified enzymes ranged from 45 to 50°C and from 7.0 to 8.0, respectively. They could hydrolyze hydroxycinnamoyl esters in a substrate-specific manner when methyl ferulate, methyl caffeate, methyl ρ-coumarate and methyl sinapinate were used as substrates. Moreover, these four FAEs were able to hydrolyze CS to release hydroxycinnamic acids. Furthermore, these strains could degrade hydroxycinnamic esters, and *L. amylovorus* CGMCC 11056 was the most efficient strain among these four isolates. These results provided a new target for the development of inoculants to improve silage quality and digestibility.

## Introduction

Lactic acid bacteria (LAB) are widely distributed in the surface of plants and fermented dairy foods, where they produce organic acids, enzymes and metabolites, contributing to the organoleptic property, flavor and long-shelf life of final products (Torino et al., 2015; Zannini et al., 2016). These bacteria also have beneficial effects on intestinal tracts in human and animals (Yu et al., 2016; Zhang et al., 2016). Moreover, LAB play important roles in silage fermentation, which depends on the production of sufficient organic acids to inhibit the growth of undesirable microorganisms, such as spoilage bacteria, food-borne pathogens, yeasts and molds, under anaerobic conditions (Li and Nishino, 2011; Dunière et al., 2013). Therefore, the tolerance to low pH and high lactic acid-producing properties have been considered as the primary criteria for the selection of LAB used as silage inoculants (Holzer et al., 2003; Ni et al., 2015).

Silage is an important feedstuff resource of ruminants worldwide, and corn stover (CS) is a very popular forage crop and widely used for ensilage in China, particularly in the north region of China (Gollop et al., 2005). CS mainly consists of cellulose, hemicellulose and lignin, all of which prevent the efficient utilization of CS in feedlots due to its low digestibility and nutrition content (Ribeiro et al., 2015). In these cases, cellulase or xylanase inoculants are often applied to agricultural stover during the ensiling process in order to degrade the cell wall polymers into water soluble carbohydrates (WSC), by which the growth of LAB can be accelerated (Sun et al., 2012). However, the action of those degradation enzymes is often limited by the presence of esterification, preventing the accessibility of depolymerases to plant cell walls. The feruloyl esters are commonly found in arabinose and xylose in plant cell walls, and they often assemble into extended networks to form an effective protective layer to the inner components of cell walls (Faulds and Williamson, 1999; Akin, 2008). Several pretreatment strategies have been made to improve the bioconversion of lignocellulosic biomass, including steam explosion, acidic or alkaline treatment. However, these pre-treatments are costly, environmentally unfriendly and unsuitable for the ensiling process (Keller et al., 2003). Therefore, feruloyl esterase (FAE), considered as an important accessory enzyme which is able to cleave the ester linkage between the ferulate and polysaccharide chain in the cell walls, is attracting more and more attention (Crepin et al., 2004; Koseki et al., 2009).

Considering their great potential in industrial and agricultural applications, most FAEs have been identified in plants and microorganisms (Ou and Kwok, 2004; Espin et al., 2016). But, until now, the well-characterized FAEs are mainly from fungi, and fewer studies have been performed in bacteria (Wong, 2006). Recently, several *Lactobacillus*-derived FAEs have been isolated from the gastrointestinal tracts or the fermentation of plant-derived food products (Couteau et al., 2001; Guglielmetti et al., 2008). Moreover, *Lactobacillus*-derived FAEs constitute an interesting group of enzymes with potential applications in the food and pharmaceutical industries due to the release of phenolic compounds from plant cell walls (Mukdsi et al., 2011; Anastasia et al., 2012). From another point of view, the *Lactobacillus* species with FAE activity could be used to develop silage inoculants due to the cleaving effects of FAE on lignocellulosic biomass, leading to improved silage quality and digestibility. However, knowledge about the *Lactobacillus*-derived FAEs is limited, and their contribution to the degradation of hydroxycinnamoyl ester in CS remains largely unexplored.

In the present study, four *Lactobacillus* strains with FAE activity were isolated from the ensiled CS, and their biochemical properties including their activity against model substrates and CS were comparatively analyzed. We aimed to improve the silage quality and digestibility using new bacterial inoculants instead of the addition of fibrolytic enzymes during the silage fermentation.

## Materials and Methods

### Bacterial Strains, Plasmids, Growth Conditions, Chemicals and DNA Manipulation

The *Lactobacillus* strains were cultured statically at 37°C in MRS (de Man, Rogosa and Sharpe) broth (Oxoid, Basingstoke, United Kingdom). *Escherichia coli* strains were cultured in LB (Luria-Bertani) medium at 37°C aerobically. *E. coli* DH5α was used for all DNA manipulations. *E. coli* BL21 (DE3) was used as the host cell for protein expression with the pET15b or pET28b vector (Liu et al., 2016; Yi et al., 2016). If necessary, ampicillin was added at a concentration of 100 μg/mL, or kanamycin at 50 μg/mL. Methyl ferulate, methyl caffeate, methyl ρ-coumarate and methyl sinapinate, used as model substrates for enzyme assays of FAE, were purchased from Sigma Chemicals Industries., Ltd. (San Francisco, CA, United States). Para-nitrophenyl ferulate (ρNPF) procured from the Industry Academy of Qilu (Shandong, China) was used as substrate for determination of FAE activity.

The genomic DNA of bacterial strains was prepared using Bacterial Genomic Extraction Kit (Tiangen, Beijing, China). The amount and purity of DNA was determined spectrophotometrically with a Biophotometer (Eppendorf, Hamburg, Germany), and then stored at -20°C until use. The genomic DNAs were used as templates to PCR amplify 16S rRNA genes and FAE genes with the primers listed in **Table 1**. All PCR reactions were performed with Ex Taq polymerase (TaKaRa, Tokyo, Japan). Restriction enzymes and T_4_ DNA ligase were also purchased from TaKaRa Biotechnology Co., Ltd. (Tokyo, Japan). Nucleotide sequences were determined by Biosune Biotechnology Co. Ltd. (Shanghai, China). Sequence similarity searches were performed using Basic Local Alignment Search Tool (BLAST).

**Table 1:** Oligonucleotide primers used in this study.

Primer	Sequence(5′-3′)	Restriction sites	Ligated vector	Template
27F	AGAGTTTGATCCTGGCTCAG			
1492R	GGTTACCTTGTTACGACTT			
P1	TCATTGCGCCAATCTTTACAAT			*L. amylovorus* CGMCC 11056
P2	CAGATTTGCGAGATTTTCATG			
P3	GATAAAATTAATCTTTCCAAT			*L. acidophilus* CCTCC AB2010208
P4	CAGATTTGCGAGACTTTCATG			
P5	CCGATGATTAAAAATTTTTCCATT			*L. farciminis* CCTCC AB2016237
P6	CGCTCAAAACGTGTGCCCGT			
P7	ACTTTGCGCCCCGCCGCTTGT			*L. fermentum* CCTCC AB2010204
P8	CTCGACTTCATTAACTTATTC			
P9	TATACATatgtcccgcattacaattg	*Nde*I	pET28b	*L. amylovorus* CGMCC 11056
P10	TATGCTCGAGctagaataatggtttt	*Xho*I		
P11	TGCGCATatgtctcgcattacaattg	*Nde*I	pET28b	*L. acidophilus* CCTCC AB2010208
P12	TATACTCGAGttaaaataggggcttc	*Xho*I		
P13	TGCGCATatgaaagtagaaattaaac	*Nde*I	pET28b	*L. farciminis* CCTCC AB2016237
P14	TATACTCGAGttaatccaacatgaat	*Xho*I		
P15	TGCGCATatggaagttgcaatcaag	*Nde*I	pET15b	*L. fermentum* CCTCC AB2010204
P16	TCGCGGATCCttagtttaagaaatcg	*Bam*HI		


*The restriction sites are underlined, and the nucleotides pairing with the potential FAE gene sequence are in lowercase letters.*

### Screening and Identification of *Lactobacillus* Species with FAE Activity

The silage samples used for screening strains were collected from a farm in Yucheng city (Shandong, China). This silage was prepared as follows: CS was chopped into about 20 mm in length, and packed into a silo without any additives, then the silo was sealed and stored at ambient temperature for 1 year. The collected CS samples (25 g) were blended with 225 mL of sterilized water for 30 min. Then, the suspension was serially diluted, spread on MRS plates with glucose omitted but containing 6.7 mM ethyl ferulate, and incubated at 37°C for 3 days. The bacteria forming a clear zone around colonies were picked out and streaked for further isolation, then the purified strains were stored at -80°C.

Morphological, physiological and biochemical characteristics of these LAB strains were determined after 12 h of incubation in MRS broth. Gram staining, catalase activity and gas production from glucose were tested. Growth at different temperatures was measured after incubation at 15, 20, 25, 30, 45, and 50°C in MRS broth for 7 days. Growth at different pH was observed after incubation at 37°C for 7 days in MRS broth with pH 3.0, 3.5, 4.0, 4.5, 8.5, and 9.5. These phenotypic characteristics provided the preliminary identification of these isolates according to the criteria of Bergey’s Manual of Determinative Bacteriology. To further identify these LAB genetically, 16S rRNA genes were amplified, sequenced and aligned. The amplification primers 27F/1492R and 454 sequencing platform were used in the sequencing. The obtained sequences were compared with sequences from other LAB strains held in the GenBank (Ni et al., 2015).

### Cloning and Expression of Putative FAEs

Based on the complete genome sequences of *L. amylovorus* GRL1112 (Kant et al., 2011), *L. acidophilus* NCFM (Altermann et al., 2005), *L. farciminis* KCTC3681 (Nam et al., 2011) and *L. fermentum* IFO3956 (Morita et al., 2008) available in GenBank (Benson et al., 2013), primers from P1 to P8 (**Table 1**) were designed to PCR amplify the putative FAE genes and the adjacent sequences with the genomic DNA of these isolates as templates. The PCR products were sequenced, and a phylogenetic tree of these putative FAEs was constructed using neighbor-joining method with MEGA5. The percentage of similarity between these sequences was calculated using BioEdit software.

Primers P9-P16 (**Table 1**) were designed to clone the putative FAE genes, then PCR products were digested with *Nde*I/*Xho*I or *Nde*I/*Bam*HI and ligated into the corresponding sites of pET28b or pET15b vector. The resultant plasmids pET-faes were transformed into *E. coli* BL21 (DE3), generating the recombinants *E. coli*/pET-faes. To prepare purified FAE protein, *E. coli*/pET-faes were grown at 37°C aerobically to an optical density (OD) at 600 nm of 0.5, then 0.5 mM isopropyl-β-D-thiogalactopyranoside (IPTG) was added to induce FAE expression for 12 h at 16°C. After purification by Ni-NTA affinity chromatography and dialysis against 50 mM sodium phosphate (pH 7.0), these purified enzymes were subjected to the SDS–PAGE gel for purification analysis. The protein quantity was estimated by the Bradford protein assay (Cheng et al., 2016).

### Biochemical Properties of FAEs

Feruloyl esterase activity was monitored at 410 nm by a spectrophotometer using ρNPF as substrate (Latha et al., 2007). In brief, a stock of 25 mM ρNPF was prepared in DMSO and mixed with 100 mM sodium phosphate buffer (pH 7.0) containing 1% Tween-80 to obtain a 1 mM substrate final concentration. The reaction mixture containing 10 μL diluted enzyme solution and 990 μL substrate solution was incubated at 37°C for 10 min. As controls, substrate solution without enzyme was utilized to account for any spontaneous hydrolysis of ρNPF. The released ρ-nitrophenol was monitored at 410 nm by a spectrophotometer. All assays were performed in triplicate. One enzyme unit was defined as the amount of enzyme required to release 1 μmol ρ-nitrophenol per minute under the experimental conditions.

The reactions were carried out in sodium phosphate buffer (100 mM, pH 7.0) at 25, 30, 35, 40, 45, 50, 55, and 60°C to determine optimum temperature for the enzyme. The thermostability was tested by measuring the residual activity with standard assay after incubating enzyme in sodium phosphate buffer (100 mM, pH 7.0) at 40 and 50°C for 5, 15, and 30 min, and 1, 2, 4, 8, and 12 h. The optimum pH was measured at 37°C using citrate-phosphate buffer (100 mM, pH 3.0–6.5), phosphate buffer (100 mM, pH 6.0–8.0) and Tris–HCl buffer (100 mM, pH 7.5–9.0). To estimate pH stability, the residual activity was measured with standard assay after preincubation in the above-mentioned buffers (pH 3.0–9.0) at room temperature for 24 h. To investigate the effect of metal ions on enzyme activity, the chloride salt of metal ions (K^+^, Ca^2+^, Mg^2+^, Fe^2+^, Fe^3+^, Mn^2+^, Cu^2+^, Zn^2+^, Ba^2+^, and Co^2+^) were added to the purified enzyme solution at the final concentration of 1 and 10 mM. After preincubating enzyme in each individual metal ion solution at 4°C for 2 h, the residual activity was measured under standard conditions. The effect of salt concentration on stability of these enzymes was studied by measuring the residual enzyme activity under standard assay conditions after preincubation of enzyme for 24 h in the presence of NaCl with different concentrations (0.1, 1, 2, 3, and 4 M) at room temperature.

### FAEs Hydrolytic Activity on Model Substrates

Four model substrates of FAEs (methyl ferulate, methyl caffeate, methyl ρ-coumarate, and methyl sinapinate) were used to measure the hydrolytic activity of purified FAEs according to the modification of a previous method (Wang et al., 2014). Briefly, the enzyme reaction was carried out in 1 mL of 100 mM sodium phosphate buffer (pH 7.0) containing 0.5 mM substrate and initialized by adding 200 ng of purified FAE. After 10 min incubation at 37°C, 1 mL 50% acetate (v/v) was added to terminate the reaction. The same reaction system with the heat inactivated enzyme was used as a control.

To avoid the interference of phenolic compounds present in MRS medium, a modified medium was prepared as described previously for the substrates degradation assays (Rozès and Peres, 1998; Esteban-Torres et al., 2015). These four strains were cultivated in a modified basal medium supplemented with filter-sterilized hydroxycinnamoyl esters at the final concentration of 1 mM. After incubation for 7 days at 37°C in darkness, the cultures were homogenized by Precellys (Bertin, Paris, France) for metabolites analysis as follows: After centrifugation at 7,500 × *g* for 5 min, the supernatant was filtered through a 0.45-μm PVDF filter. The residual hydroxycinnamic ester and the generated hydroxycinnamic acid in supernatant was detected by High Performance Liquid Chromatography (HPLC) with an XBridge BEH300 C18 reverse phase column (150 mm × 4.6 mm; Waters, Milford, United States) at a flow rate of 1.0 mL/min. The HPLC system (Shimadzu, Kyoto, Japan) was equipped with a SCL-10A system controller, a LC-10AT pump, a SIL-10AD auto-injector and a SPD-M10A diode array detector. The separation of substrate and product was effected with solvent A (methanol) and solvent B (water and acetic acid, 99:1, v/v) at a ratio of 1:1. The column eluent was monitored at the A_320_.

### Release of Hydroxycinnamic Acids from CS

Fresh CS was collected from Yucheng city (Shandong, China). Dry matter (DM) of the fresh materials was analyzed according to the drier drying determination method at 65°C for 48 h (Sun et al., 2012). WSC was measured by the phenol sulphuric acid method (Dubois et al., 1956). Neutral detergent fiber (NDF), acid detergent fiber (ADF) and lignin were determined with the Fibertec 2010 (Foss, Copenhagen, Denmark). The crude protein (CP) was determined with Kjeldahl Nitrogen Determination Method (Krishnamoorthy et al., 1982). The CS was chopped into 20 mm around in length and washed twice with sterilized water. Hydrolysis of CS by 1 mg purified FAE was carried out in 40 mL of 100 mM sodium phosphate buffer (pH 7.0) containing 4 g CS. After incubation at 37°C for 24 h, the reaction was terminated by boiling for 5 min. After vortexing and centrifugation, the supernatant was recovered and filtered. Free hydroxycinnamic acids in supernatant were analyzed by HPLC as described above.

### Nucleotide Sequence Accession Numbers

The sequences in the present study have been submitted to the GenBank nucleotide sequence database, and obtained the accession number as follows: MF045127 for the 16S rRNA gene of *L. amylovorus* CGMCC 11056, MF045128 for *L. acidophilus* CCTCC AB2010208, MF045129 for *L. farciminis* CCTCC AB2016237, and MF045130 for *L. fermentum* CCTCC AB2010204. Four FAEs are under accession numbers KX545370 (*faeLam*), KX545371 (*faeLac*), KX545372 (*faeLfa*) and KX545373 (*faeLfe*), respectively.

## Results

### Isolation and Identification of Strains with FAE Activity

**FIGURE 1:**
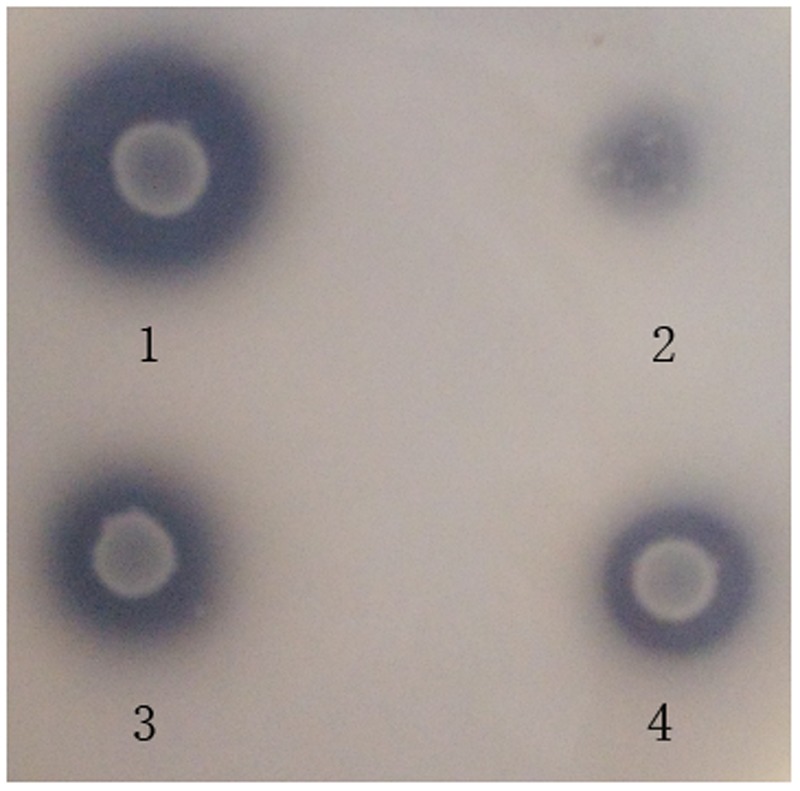
**Plate screening assay showing FAE activities by clear zones around the individual colonies.** The plates were incubated at 37°C for 72 h. 1 is an *L. amylovorus* CGMCC 11056 colony. 2 is an *L. acidophilus* CCTCC AB2010208 colony. 3 is an *L. farciminis* CCTCC AB2016237 colony. 4 is an *L. fermentum* CCTCC AB2010204 colony.

Among all the isolates derived from ensiled CS, 4 of them showed clear zones around the colonies on the MRS agar containing synthetic phenolic ester ethyl ferulate (**Figure 1**), indicating that these isolates were able to hydrolyze the hydroxycinnamoyl ester, and were FAE-producing strains. The morphological and physiological characteristics of these isolates are shown in **Table 2**. All strains were Gram-positive and catalase-negative bacteria, and able to grow at 25–45°C and at pH 4.0–9.5. To identify these isolates at species level, their 16S rRNA genes were sequenced and aligned, which showed 100, 99, 99, and 100% identity with *L. amylovorus* 14LAB1, *L. acidophilus* NCFM, *L. farciminis* KCTC3681 and *L. fermentum* IFO3956, respectively. Based on these phenotypic characteristics, combined with the 16S rDNA sequences, these four isolates were identified as *L. amylovorus*, *L. acidophilus*, *L. farciminis* and *L. fermentum.* These strains were deposited in the China General Microbiological Culture Collection Center (CGMCC) or China Center for Type Culture Collection (CCTCC), and named as CGMCC 11056, CCTCC AB2010208, CCTCC AB2016237, and CCTCC AB2010204, respectively.

**Table 2:** Phenotypic characteristics of the four FAE-producing strains isolated from ensiled CS.

	*L. amylovorus* CGMCC 11056	*L. acidophilus* CCTCC AB2010208	*L. farciminis* CCTCC AB2016237	*L. fermentum* CCTCC AB2010204
Shape	Rod	Rod	Rod	Rod
Gram stain	+	+	+	+
Catalase	-	-	-	-
Gas from glucose	-	-	-	+
Fermentation type	Homo	Homo	Homo	Hetero
**Growth at temperature (°C)**
15	-	-	+	w
20	-	-	+	+
25	+	w	+	+
30	+	+	+	+
45	+	+	W	w
50	+	-	-	w
**Growth at pH**
3.0	-	-	-	w
3.5	w	-	w	+
4.0	+	w	+	+
4.5	+	+	+	+
8.5	+	+	+	+
9.5	+	+	+	+


*Homo, Homofermentative; Hetero, Heterofermentative; +, positive; w, weak positive; -, negative.*

### Cloning of FAE Genes and Diversity Analysis

**FIGURE 2:**
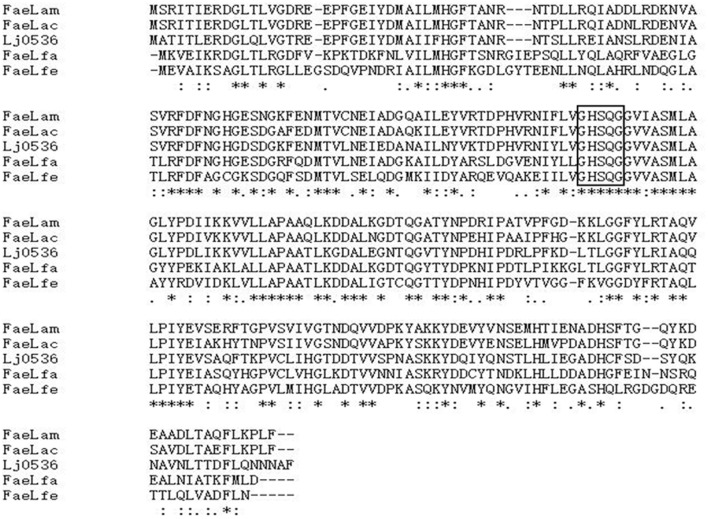
**Multiple sequence alignment of FaeLam, FaeLac, FaeLfa, FaeLfe, and Lj0536, with the classical serine esterase catalytic motif GXSXG in the box.** Lj0536 is an FAE derived from *L. johnsonii* N6.2. Asterisk indicates that amino acid residues are conserved in all proteins. The proteins of FaeLam, FaeLac, FaeLfa and FaeLfe showed sequence identity in the range of 45–85%, and sequence identity with Lj0536 from 44 to 71%. Alignment was done using Clustal W with the algorithm of Myers and Miller, and the percentage of similarity was calculated using BioEdit software.

Based on the whole genome sequences available in GenBank, primer sets specific to these FAEs were designed to PCR amplify FAE genes from the corresponding genomes of these four *Lactobacillus* strains. Four putative FAE genes with almost the same size (744–750 bp) were cloned from *L. amylovorus* CGMCC 11056, *L. acidophilus* CCTCC AB2010208, *L. farciminis* CCTCC AB2016237 and *L. fermentum* CCTCC AB2010204. They were named *faeLam*, *faeLac*, *faeLfa*, and *faeLfe*, respectively. Their deduced amino acid sequences shared identity in the range of 45–85%, and they had identity from 44 to 71% with Lj0536, a well-characterized FAE isolated from *L. johnsonii* N6.2. (Lai et al., 2009). Moreover, a conserved motif (Gly-x-Ser-x-Gly) required for FAE activity was observed in these proteins, suggesting that these four *Lactobacillus* species were FAE-producing strains (**Figure 2**).

**FIGURE 3:**
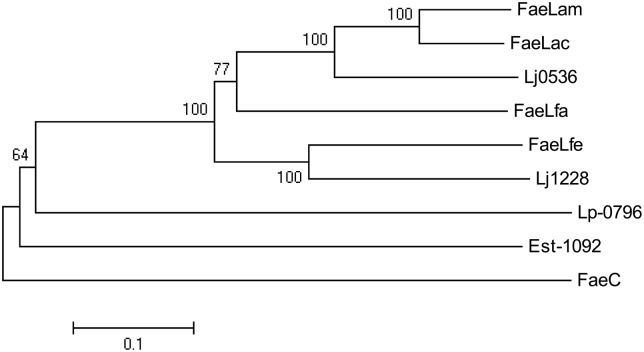
**Phylogenetic tree based on amino acid sequences of FaeLam, FaeLac, FaeLfa and FaeLfe and other reported FAEs cloned from *Lactobacillus* species, including Lp-0796 from *L. plantarum* WCFS1, Est-1092 from *L. plantarum* DSM1055, Lj1228 and Lj0536 from *L. johnsonii* N6.2.** FaeC from *Aspergillus niger* N402 was used as an outgroup. The tree was constructed using MEGA5 with the neighbor-joining algorithm. Bootstrap values for 1000 replicates are shown at the node of the tree. The bar indicates 10% sequence divergence.

The phylogenetic tree was depicted with the reported FAE sequences derived from *Lactobacillus* species, including Lp-0796 from *L. plantarum* WCFS1 (Esteban-Torres et al., 2013), Est-1092 from *L. plantarum* DSM1055 (Esteban-Torres et al., 2015), Lj1228 and Lj0536 from *L. johnsonii* N6.2 (Lai et al., 2009). FaeC, a FAE from *Aspergillus niger* N402, was used as an outgroup (Dilokpimol et al., 2017). As shown in **Figure 3**, these FAEs of *Lactobacillus* could be divided into several classes. The proteins of FaeLam, FaeLac, FaeLfa and Lj0536 grouped into the same cluster. Others contained the proteins of FaeLfe and Lj1228. These proteins have distant phylogenetic relationships with Est-1092 and Lp-0796, the FAEs derived from the two *L. plantarum* strains.

### Biochemical Properties of Isolated FAEs

**FIGURE 4:**
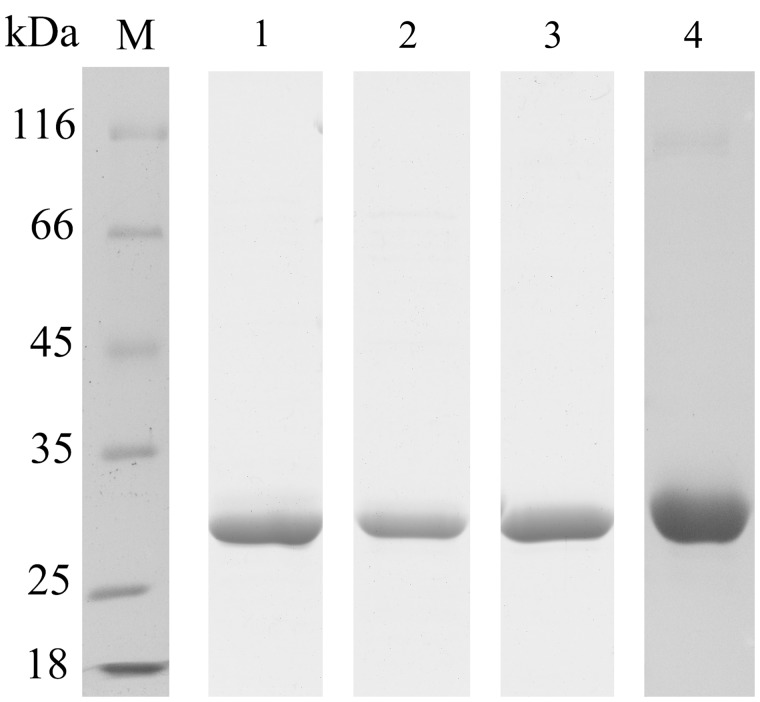
**Purification of recombinant FAEs.** Proteins were examined by 12% SDS–PAGE. Lane M, protein molecular-weight markers; lane 1, purified FaeLam; lane 2, purified FaeLac; lane 3, purified FaeLfa; lane 4, purified FaeLfe.

To prepare FAE proteins for biochemical analysis, these four FAE genes were hetero-expressed in *E. coli* BL21 (DE3) and purified by Ni-column. The eluted proteins were then dialyzed against sodium phosphate buffer (50 mM, pH 7.0) to remove the imidazole, which may interfere with FAE activity assays. As shown in **Figure 4**, four purified proteins of approximately 29 kDa were separated by SDS–PAGE gel to estimate the molecular mass of each FAE of these four strains.

**FIGURE 5:**
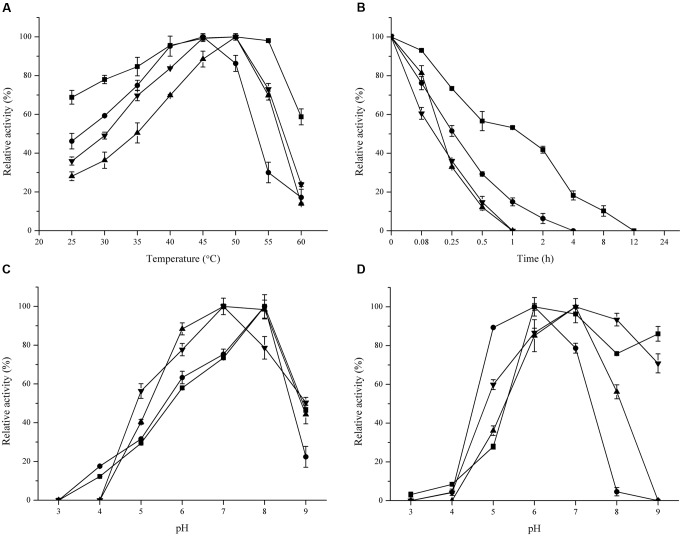
**Effect of temperature and pH on activity and stability of FaeLam (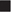), FaeLac (•), FaeLfa (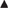) and FaeLfe (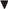).** For each FAE, The observed maximum activity was defined as 100%. **(A)** The reactions were performed in phosphate buffer (100 mM, pH 7.0) at different temperatures to measure optimum temperature. **(B)** Thermostability was determined by measuring the residual activity after incubating enzyme in 50°C for different periods of time. **(C)** The optimum pH was identified at 37°C using 3 buffer systems (citrate–phosphate, phosphate and Tris–HCl). **(D)** The enzymes were preincubated in buffers with different pH for 24 h to test their stability.

The optimal temperature of these FAEs ranged from 45 to 50°C. Unexpectedly, FaeLam kept 60% residual activity at 60°C, while the other FAEs lost most of their activity at this temperature (**Figure 5A**). All enzymes were stable at 40°C (data not shown). When temperature was increased to 50°C, the half-life of FaeLam and FaeLac were 1 h and 15 min, respectively, while no activity was detected with the other FAEs after 60 min treatment (**Figure 5B**). The optimal pH for these four FAEs activities were investigated. As shown in **Figure 5C**, the optimal pH value was from 7.0 to 8.0, and the activities dropped drastically at pH > 8.0 or <6.0. Interestingly, these four esterases displayed different pH stabilities. FaeLam and FaeLfe were stable at pH range of 6.0–9.0 with over 75% residual activity, while over 80% activity retained at pH 5.0–7.0 for FaeLac and FaeLfa (**Figure 5D**). These results indicated that the FAEs from the *Lactobacillus* strains have different optimal reaction conditions.

**FIGURE 6:**
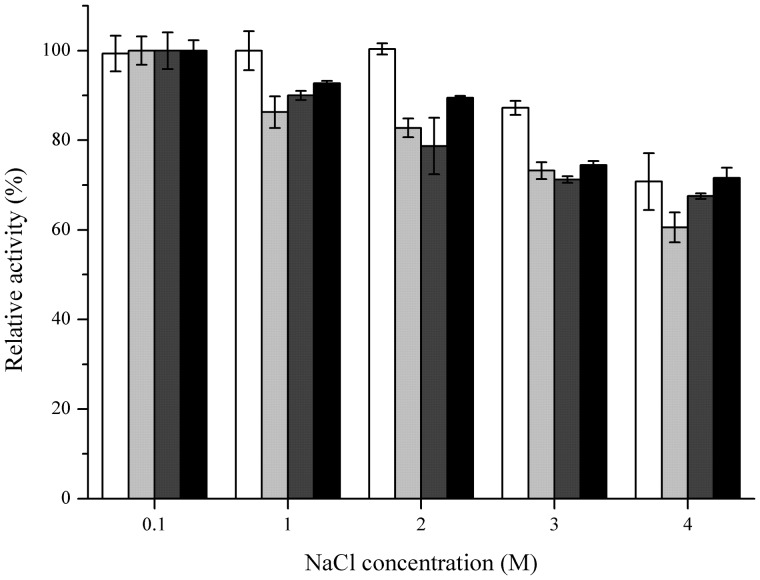
**Effect of NaCl concentration on stabilities of FaeLam (white bar), FaeLac (light gray bar), FaeLfa (dark gray bar) and FaeLfe (black bar).** For each FAE, The observed maximum activity was defined as 100%. The stability was determined after preincubation of enzyme for 24 h in the presence of NaCl at different concentrations.

Several metal ions could inhibit the activities of these four FAEs (**Table 3**), including Fe^2+^, Fe^3+^ and Cu^2+^, particularly at the high concentration (10 mM). Moreover, FaeLam could tolerate 10 mM Zn^2+^ with 67% activity remaining. These four FAEs retained about 70% of their activity in the presence of 4 M NaCl, indicating that they could be classified as halotolerant enzymes (**Figure 6**).

**Table 3:** Effect of various metal ions on relative activity of the FAEs.

Metal ions	Relative activity (%)							
	
	FaeLam		FaeLac		FaeLfa		FaeLfe	
				
	1 mM	10 mM	1 mM	10 mM	1 mM	10 mM	1 mM	10 mM
K^+^	99.2 ± 4.3	84.4 ± 4.3	97.5 ± 1.9	78.5 ± 10.8	102.8 ± 2.4	91.9 ± 0.2	90.3 ± 1.4	74.9 ± 2.3
Ca^2+^	99.9 ± 0.7	86.6 ± 8.7	99.5 ± 1.6	89.8 ± 1.9	101.9 ± 4.3	82.7 ± 10.2	90.3 ± 0.3	79.1 ± 0.2
Fe^2+^	57.1 ± 5.2	0	64.8 ± 13.3	5.5 ± 0.6	18.6 ± 4.4	3.6 ± 1.1	24.3 ± 2.8	0
Fe^3+^	75.6 ± 13.1	0	75.7 ± 0.4	0	40.1 ± 8.5	1.8 ± 0.2	62.5 ± 2.8	0
Mg^2+^	103.9 ± 1.5	84.6 ± 2.0	93.6 ± 0.4	90.6 ± 0.7	100.1 ± 1.1	93.8 ± 1.0	104.6 ± 6.4	82.3 ± 0.7
Cu^2+^	63.6 ± 7.1	0	23.1 ± 4.5	3.4 ± 0.5	5.3 ± 0.5	2.12 ± 0.1	0	0
Zn^2+^	88.0 ± 5.6	67.5 ± 1.3	84.9 ± 2.3	18.7 ± 0.3	103.1 ± 0.9	11.2 ± 0.1	91.4 ± 4.1	0.9 ± 0.3
Ba^2+^	101.7 ± 6.3	82.2 ± 1.8	96.2 ± 0.5	82.1 ± 1.2	101.4 ± 1.8	84.8 ± 0.1	104.2 ± 3.2	68.5 ± 5.1
Mn^2+^	98.5 ± 10.2	66.2 ± 4.6	93.2 ± 3.3	77.7 ± 0.5	101.3 ± 2.4	82.6 ± 1.4	103.2 ± 4.6	70.5 ± 1.8
Co^2+^	93.1 ± 0.5	74.0 ± 4.6	106.2 ± 5.9	85.2 ± 7.9	96.5 ± 0.4	85.9 ± 0.5	101.7 ± 7.8	88.8 ± 1.0


*FAE activity in the absence of metal ions was regarded as 100%.*

### Degrading Ability of These FAEs against Model Substrates

The activity of purified FAEs against four model substrates was determined. The results showed that all of these FAEs could efficiently hydrolyze hydroxycinnamoyl esters. However, these four enzymes displayed different preference to the tested substrates. FaeLfa had greater activity on methyl ferulate, methyl caffeate and methyl ρ-coumarate, while FaeLam had the highest degradation efficiency against methyl sinapinate. These four enzymes all showed the highest hydrolytic activity against methyl caffeate, and the lowest against methyl sinapinate, indicating these four FAEs have the same preference for substrates (**Table 4**).

**Table 4:** Conversion rates of the four FAEs on model substrates.

Substrates	Conversion rate (%)			
	
	FaeLam	FaeLac	FaeLfa	FaeLfe
Methyl ferulate	13.6 ± 0.9	5.8 ± 0.5	27.2 ± 1.2	16.0 ± 0.5
Methyl caffeate	70.6 ± 2.3	37.1 ± 0.9	95.0 ± 2.1	73.2 ± 1.7
Methyl ρ-coumarate	16.6 ± 1.1	8.0 ± 0.7	34.0 ± 1.6	13.0 ± 0.5
Methyl sinapinate	12.4 ± 0.6	2.6 ± 0.3	4.6 ± 0.3	2.8 ± 0.1


*The reaction was performed at 37°C for 10 min with 0.5 mM substrate and 200 ng of purified FAE.*

To further investigate the abilities to hydrolyze hydroxycinnamoyl esters, these four isolates were grown in a modified medium containing model substrates. The concentration of hydroxycinnamoyl esters and hydroxycinnamic acids in the medium was measured by HLPC. The results showed that these four *Lactobacillus* strains could efficiently hydrolyze the model substrates except *L. farciminis* CCTCC AB2016237 and *L. fermentum* CCTCC AB2010204 which showed low hydrolytic activities to methyl sinapinate (**Table 5**). Interestingly, these four strains also exhibited the ability to metabolize hydroxycinnamic acids because only a low amount of hydroxycinnamic acids was left in the cultures, particularly in the culture of *L. amylovorus* CGMCC 11056.

**Table 5:** The concentration (μM) of hydroxycinnamoyl esters and corresponding hydroxycinnamic acids in the cultures.

	*L. amylovorus* CGMCC 11056	*L. acidophilus* CCTCC AB2010208	*L. farciminis* CCTCC AB2016237	*L. fermentum* CCTCC AB2010204
Methyl ferulate	19.1 ± 2.3	16.5 ± 1.2	76.6 ± 4.5	17.4 ± 1.6
Ferulic acid	8.7 ± 2.9	704.2 ± 29.8	641.1 ± 21	193.6 ± 16.8
Methyl caffeate	11.5 ± 1.5	9.9 ± 2.7	14.0 ± 1.8	11.9 ± 0.9
Caffeic acid	108.6 ± 7.3	432.0 ± 19.1	102.8 ± 5.4	141.8 ± 9.4
Methyl ρ-coumarate	11.0 ± 0.5	10.7 ± 1.3	35.9 ± 4.2	10.8 ± 0.9
ρ-Coumaric acid	5.6 ± 1.3	629.3 ± 25.4	770.5 ± 28.7	170.9 ± 13.3
Methyl sinapinate	23.1 ± 3.6	16.7 ± 1.1	586.5 ± 21.9	251.7 ± 16.6
Sinapic acid	54.2 ± 4.7	638.8 ± 21.3	233.4 ± 14.8	592.1 ± 22.7


*The initial concentration of hydroxycinnamoyl esters was 1 mM. After incubated these four isolates at 37°C for 7 days, the residual hydroxycinnamoyl esters and the generated hydroxycinnamic acids in the bacterial cultures were detected.*

### Enzymatic Hydrolysis of CS

**FIGURE 7:**
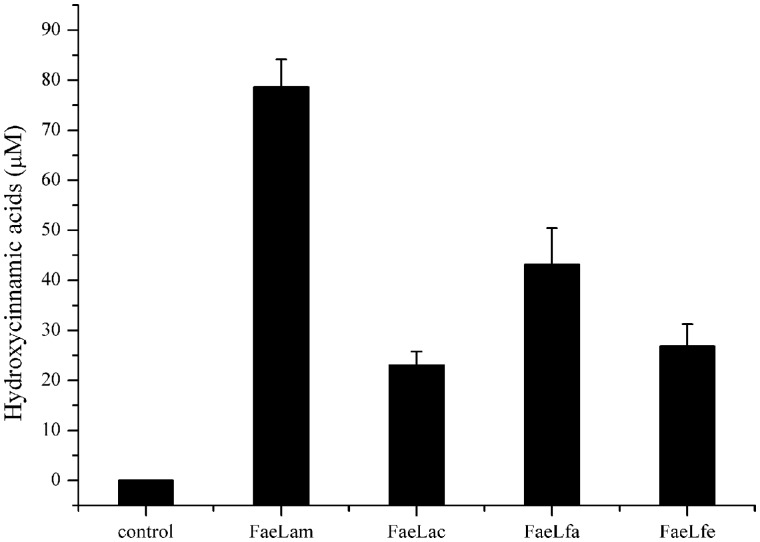
**The release of hydroxycinnamic acids from CS treated with FaeLam, FaeLac, FaeLfa and FaeLfe.** Hydrolysis of 4 g CS by 1 mg of each purified FAE was carried out in 40 mL phosphate buffer (100 mM, pH 7.0) and then incubated at 37°C for 24 h. Free hydroxycinnamic acids in the supernatant were analyzed by HPLC.

CS is mainly composed of cellulose, hemicellulose and lignin which limited the efficient utilization of the stover silages. **Table 6** shows the chemical composition of the fresh CS used in this study. To evaluate the degradation ability of these four isolates to CS, we inoculated these four strains to the chopped CS medium and cultured them for 72 h at 37°C to determine the release of the hydroxycinnamic acids, however, no hydroxycinnamic acids were detected in their media (data not shown). Subsequently, each of these four purified esterases was mixed with the chopped CS at 37°C for 24 h. As shown in **Figure 7**, these FAEs could hydrolyze the CS to release hydroxycinnamic acids, but the hydrolytic abilities of the enzymes were different. FaeLam displayed the highest hydrolytic activity with release of 80 μM hydroxycinnamic acid, while the lowest activity was obtained with FaeLac. These results indicated that these four enzymes were able to hydrolyze the esters between hydroxycinnamic acids and xylose or arabinose in the cell walls of CS, and the hydrolytic activity on CS was strain-specific.

**Table 6:** Chemical composition of the fresh CS.

Chemical composition	Percentage (%)
DM	23.4 ± 0.6
WSC	4.3 ± 0.3
NDF	65.8 ± 2.0
ADF	35.7 ± 0.4
CP	3.7 ± 0.3
Lignin	14.2 ± 1.2


*DM, dry matter; WSC, water-soluble carbohydrates; NDF, neutral detergent fiber; ADF, acid detergent fiber; CP, crude protein. WSC, NDF, ADF, CP and Lignin were calculated as the percentage of DM.*

## Discussion

As a subclass of carboxylesterase, FAE (EC 3.1.1.73) acts as an accessory enzyme to disrupt plant cell walls, making agricultural wastes more easily accessible for animal feed processing (Wong, 2006). *In situ* digestibility studies revealed that complete or partial hydrolysis of ferulic acid linkages in the cell walls could directly improve ruminal digestion or increase the susceptibility of cell walls to ruminal digestion. Therefore, more attention has been paid to FAE, which is a new feedlot enzyme with valuable application for ensiling (Aboagye et al., 2015). So far, FAEs are mainly produced from fungi, such as *A. niger* (de Vries et al., 2002), and these fungi are strictly aerobic microorganisms, which cannot be grown under anaerobic condition, including the ensiling process. In addition, exogenous addition of enzyme results in increased silage costs. In those cases, LAB with FAE activity as bacterial inoculants could promote the lactic acid production as well as fiber degradation in the silage fermentation. In this study, four FAE-producing *Lactobacillus* species (*L. amylovorus* CGMCC 11056, *L. acidophilus* CCTCC AB2010208, *L. farciminis* CCTCC AB2016237, and *L. fermentum* CCTCC AB2010204) were isolated from naturally fermented silages. The biochemical properties showed that these four isolates and their produced FAEs had a broad range of hydrolytic activity against the hydroxycinnamoyl esters tested here, and hydroxycinnamic acids could be released from CS by these enzymes. These findings suggested that these *Lactobacillus* species have the potential for development of new silage inoculants, which might not only improve silage quality, but also contribute to the probiotic effects on animals.

Biochemical properties of FAEs from these four isolates revealed that the optimum temperature, pH and metal ions of these esterases were different from strain to strain although they were isolated from the same silage environments (Udea et al., 2014). These results are in agreement with findings with FAEs Lj1228 and Lj0536 isolated from the same species *L. johnsonii* (Lai et al., 2009). Also these four FAEs were stable at higher temperature, particularly FaeLam, which retained 50% activity after 1 h at 50°C. This result was similar to that of FAE PeFaeA from the fungus *Pleurotus eryngii* (Nieter et al., 2014). Surprisingly, the optimal pH of these four esterase activities ranged from 7.0 to 8.0, although these enzyme-producing strains were isolated from acidic niches (**Figure 5C**). This finding was also found with other *Lactobacillus* FAEs (Sawatari and Yokota, 2007; Fritsch et al., 2017). In order to clarify why the optimal pH of *Lactobacillus*-derived FAEs was slightly alkaline, we examined the intracellular and extracellular FAE activities of *L. amylovorus* CGMCC 11056. Surprisingly, the FAE activity was only detected in the cytoplasm (data not shown), which was evidenced by the fact that no signal peptide motif was identified at the N-terminus of the deduced amino acid sequence of the FaeLam using SignalP 4.1 server. Therefore, the mechanism by which these strains degrade the hydroxycinnamic ester substrates or transport these substrates into the cytoplasm needs further study.

Our data demonstrated that these four FAEs could efficiently hydrolyze methyl ferulate, methyl caffeate, methyl ρ-coumarate and methyl sinapinate under the experimental conditions, suggesting that they belonged to the type C FAE family (Moukouli et al., 2008). Moreover, these FAEs exhibited the substrate preference in a strain specific manner. When these four isolates were cultured in the medium containing four model hydroxycinnamoyl esters, different rates of release of hydroxycinnamic acids were observed. However, it is worth further investigating whether the hydrolytic properties of these four strains are due, exclusively, to the activity of the characterized FAEs since some strains, such as the *L. johnson* N6.2 (Lai et al., 2009), can produce more than one FAE. Furthermore, the hydroxycinnamic acids could be liberated from CS after treatment with these FAEs. However, the content of hydroxycinnamic acid did not increase when CS was inoculated with these four strains (data not shown). It seemed likely that these strains could utilize the hydrolysates, such as hydroxycinnamic acids, and produce other metabolites.

Taken together, we isolated four *Lactobacillus* strains with FAE activity and characterized their biochemical properties in the present study. Hydroxycinnamic acids commonly occur in plant cell walls, leading to restricted digestion of ensiled CS. Hydrolysis of the esters could render the cross-linked polysaccharides more fragile and susceptible to ruminal degradation. Therefore, FAE-producing *Lactobacillus* strains and their enzymes can play crucial roles in the development of silage inoculants.

## Author Contributions

ZX performed the experiments, analyzed the data, and drafted the manuscript. HH and SZ participated in the preparation of the experimental materials. JK and TG designed the works and revised the manuscript. All authors read and approved the final manuscript.

## Conflict of Interest Statement

The authors declare that the research was conducted in the absence of any commercial or financial relationships that could be construed as a potential conflict of interest.
